# Theory of mind deficits in non-fluent primary progressive aphasia

**DOI:** 10.1016/j.cortex.2025.03.012

**Published:** 2025-04-08

**Authors:** Eleni Peristeri, Stephanie Durrleman, Sokratis Papageorgiou, Constantin Potagas, Christos Frantzidis, Anastasios Kotrotsios, Nikolaos Scarmeas, Kyrana Tsapkini

**Affiliations:** a Department of English Studies, Aristotle University of Thessaloniki, Thessaloniki, Greece; b Department of Linguistics, University of Fribourg, Fribourg, Switzerland; c Department of Neurology, School of Medicine, National and Kapodistrian University of Athens, Athens, Greece; d School of Engineering and Physical Sciences, University of Lincoln, United Kingdom; e School of Medicine, University of Thessaly, Larissa, Greece; f Department of Neurology, Columbia University, New York, NY, USA; g Department of Neurology, Johns Hopkins University, School of Medicine, Baltimore, MD, USA; h Department of Cognitive Science, Johns Hopkins University, Baltimore, MD, USA

**Keywords:** Primary progressive aphasia, Theory of mind, Syntax, Executive functions

## Abstract

Theory of Mind (ToM) is a complex socio-cognitive subdomain that is under-researched in neurodegenerative disorders, particularly in persons with primary progressive aphasia. We studied 14 persons with non-fluent/agrammatic variant primary progressive aphasia (nfaPPA), and asked two questions: (1) whether persons with nfaPPA have intact or impaired ToM, with emphasis on their false belief attribution abilities, relative to healthy controls; and (2) whether false-belief attribution (a component of ToM) is associated with their syntactic and executive function (EF) abilities. False belief understanding was tested through nonverbal videos, with participants deciding whether the story ending was an appropriate end of each video scenario or not. Syntactic production abilities were measured through repetition of syntactically simple and complex sentences (comprising length-matched complement and adjunct sentences), and EF tasks, specifically, a digit-back and an attention-shifting task. Persons with nfaPPA were less accurate than controls in adapting their reasoning to the false beliefs of other agents in the nonverbal videos of the false belief attribution task. Their false belief attribution performance was significantly predicted primarily by their syntactic production, followed by their EF. The overall findings suggest that persons with nfaPPA may have impaired performances in ToM tasks, due to impairments in basic non-social cognitive functioning, such as syntactic and EF abilities.

## 1. Introduction

Theory of Mind (ToM) is the ability to reason about mental states of oneself and others', and thus grasp that other individuals can have different thoughts, intentions and emotions from one’s own (Frith & Frith, 2005; McDonald & Kelly, 2023; Quesque et al., 2024). ToM abilities thus enable understanding, explaining and predicting others' behaviors, which promotes successful social interaction. False belief attribution has been one of the most fundamental aspects of ToM, and it refers to one’s ability to understand and act upon an agent’s point of view, even when that point of view differs from the observer’s (false belief) (Leslie et al., 2004).

ToM appears to be a multilevel socio-cognitive construct that relies on a complex balance of underlying mechanisms. Syntax has been considered to be an essential component of verbal ToM since it allows one’s thoughts of others' beliefs, intentions and emotions to be expressed in embedded propositions. In fact, verbal ToM has been particularly linked to subordination in syntax, which allows one propositional meaning to be embedded within another (De Villiers, 1998). Complement clauses, which are introduced with mental state verbs (e.g., Mary *believes* that it rains outside), can be used to articulate a false complement while the sentence as a whole remains true, since the truth value of the subordinate clause is independent of the truth or falsity of the superordinate clause. In that sense, complement syntax provides individuals with the unique linguistic means of embedding linguistic information that represents another person’s point of view, and is thus necessary for the representation of false beliefs. It follows that impairments in complement clauses would be associated with impairments in verbal ToM (De Villiers, 1998).

Executive functions (EF) have also been shown to play an important role in individuals' mentalizing abilities. Reasoning through a ToM task requires the maintenance, retrieval, and manipulation of information within working memory (WM), inhibition of competing interpretations, and attention. More specifically, in a false belief attribution task, individuals are required to make and report a decision about another person’s mental state while they know the real situation, which happens to be different from the other person’s false belief. This means that the individual needs to inhibit her/his belief about reality in order to reply from the perspective of another. Furthermore, the individual needs to manipulate the information available in order to reconstruct and compare both the real state of the world and others' mental representation of reality, which is claimed to place demands in WM (Bernstein et al., 2011).

Research in healthy controls (Qureshi et al., 2010) using a dual-task paradigm (the primary and secondary task tapping into ToM and EF, respectively) found that ToM processing costs increased with enhanced demands on the individuals' inhibition and attention shifting abilities, implying that ToM and EF are interdependent mechanisms. Bradford et al. (2015) also found positive correlations between healthy adults' performance in a ToM and a nonverbal Stroop-like task, further corroborating the links between ToM and EF. Previous research has also highlighted relations between WM and ToM reasoning in healthy controls (German & Hehman, 2006; McKinnon et al., 2007; Newton & de Villiers, 2007), offering converging evidence for the interdependence between ToM and EF abilities in language-unimpaired adults. However, we should note that due to the fact that dual tasks are, in general, resource demanding, it is possible that these effects have been found on a task, rather than a function level.

Deficits in false belief attribution have been amply documented in traumatic brain injury (Rowley et al., 2017) and other neurological conditions (Harrington et al., 2005; Hennion et al., 2016; Stone et al., 2003), as well as in neurodevelopmental syndromes with syntactic impairments (Durrleman & Franck, 2015; Nilsson & López, 2016; Yirmiya et al., 1998). Understanding that behavior is dependent on beliefs about reality rather than on reality itself is a prerequisite for functioning normally in the social world. For this reason, neuropsychological research on ToM reasoning has focused on adult populations with progressive diseases, such as the behavioral variant of frontotemporal dementia or persons with Alzheimer’s disease (e.g., Gregory et al., 2002; Le Bouc et al., 2012; Youmans & Bourgeois, 2010) that are often characterized by a progressive deterioration of social abilities (Bediou et al., 2009; McKhan et al., 2001). False belief tasks have the participant navigate through the mind of another individual by detecting their mistaken belief about the state of the world, and are sensitive in detecting ToM impairments in both neurodegenerative (see Setién-Suero et al., 2022 for a systematic review) and neurodevelopmental conditions (Baron-Cohen, 2000).

ToM deficits have only been occasionally examined in language studies with neurodegenerative populations with predominant language deficits, such as primary progressive aphasia (PPA). More, specifically, we are aware of only five studies on ToM in persons with PPA [Dodich et al., 2021; Cummings, 2019; Hazelton et al., 2017; Couto et al., 2013; Rankin et al., 2009; see Fittipaldi et al. (2019) for a review on social cognition deficits in PPA]. These studies have tested the following aspects of ToM: intention attribution (Couto et al., 2013), usage of ToM-related terms in narration (Cummings, 2019), emotion attribution (Dodich et al., 2021), perspective-taking (Hazelton et al., 2017), and social and emotion inferencing (Rankin et al., 2009). These studies form an important foundation for ToM investigations in PPA, and demonstrate a deficit in attributing mental states to other agents. Nevertheless, three of them used heavily verbal contexts (Cummings, 2019; Hazelton et al., 2017; Rankin et al., 2009), one concentrated on emotion recognition (Couto et al., 2013), and only one compared intention versus emotion recognition in nonverbal contexts (Dodich et al., 2021). None of these studies has tested false belief attribution or its relation with syntax or/and EF in PPA. Furthermore, none has tested the intriguing hypothesis laid out here that performance in false belief attribution in PPA may be particularly associated with a syntactic processing deficit in complement clauses, because the latter represent the linguistic means of attributing another person’s point of view. Therefore, we do not know how the false belief attribution system may be compromised under a syntactic and/or an EF impairment that is very common in PPA (Basaglia-Pappas et al., 2023; Butts et al., 2015; Coemans et al., 2022; Harris et al., 2019; Macoir et al., 2017; Ramanan et al., 2023).

In the present study, syntactic ability in the persons with nfaPPA was tested through a sentence repetition task. Sentence repetition has been suggested to be a useful proxy for PPA persons' WM resources, since relevant studies have found that reduced performance in sentence repetition in PPA is related with increased sentence length (Arslan et al., 2022), or low performance in digit span tasks (Beales et al., 2019; Foxe et al., 2013). Interestingly, previous research has found inconsistencies between levels of impairment across sentence repetition and digit span tasks in persons with PPA, further implying additional deficits that may be responsible for the individuals' impaired performance in sentence repetition. For example, Leyton et al. (2014) found that persons with logopenic PPA exhibited worse performance in sentence repetition than persons with nfaPPA, though digit span performance was similar across the two groups. This discrepancy was interpreted in terms of the linguistic demands imposed by sentence repetition and the fact that the ability to repeat sentences is also dependent on an individual’s morphosyntactic knowledge, in the sense that linguistic structures of high structural complexity (like complex subordinate clauses) may increase the processing effort involved in sentence repetition. Studies with persons with PPA note that syntactically complex constructions add additional processing difficulties during the task of repeating sentence material (Arslan et al., 2022; Hohlbaum et al., 2018; Macoir et al., 2021). In the current study, the sentence repetition task included both structurally simple and complex sentences, which were matched for length and lexical frequency, to allow us to disentangle effects of parsing structural complex language and WM constraints on the repetition performance of the persons with nfaPPA.

EF abilities in the present study were assessed through a two digit-back monitoring and updating WM task, as well as a global-local attention shifting task. The particular tasks measure the use of established components of EF, namely shifting and updating in the nonverbal modality, i.e., without requiring verbal output (based on Miyake et al.’s seminal factor model of ‘unity and diversity’; Miyake et al., 2000).

In the present study, we evaluated ToM abilities in PPA with a nonverbal task that tests false belief attribution, the latter being widely considered as one of the backbone aspects of the ToM construct. We used a nonverbal false belief attribution task to attenuate the potentially confounding effects of language processing demands on the ToM performance of the persons with nfaPPA.

We investigated: (i) whether the persons with nfaPPA would differ from the language-unimpaired group in false belief attribution, and (ii) the links between ToM, syntax and EF abilities in the same persons. We hypothesized that: (i) false belief attribution would be impaired in nfaPPA, along with syntax and EF, (ii) false belief attribution impairment would be associated with syntax and EF performance in the persons with nfaPPA.

## 2. Materials and methods

### 2.1. Participants

Study participants comprised a group of nfaPPA and a group of healthy matched controls. We recruited 14 persons with nfaPPA (21 persons were initially screened; nine women in the final sample, mean age in years = 64.1 ± 5.0), (mean years of education = 15.2 ± 1.7). Mean age of onset was 61 years old with mean 2.5 years disease duration at testing. The control group consisted of 20 age- and education-matched, language-unimpaired, individuals (14 women, mean age in years = 62.4 ± 3.9), (mean years of education = 14.0 ± 2.5).

We now report all inclusion/exclusion criteria, whether inclusion/exclusion criteria were established prior to data analysis, all manipulations, and all measures in the study. The persons with nfaPPA were recruited from among referrals to specialized cognitive disorders clinics and inpatient facilities of the Aiginition University Hospital in Athens, with a population estimated at 3.75 million. Symptom onset ranged from two to three years. All participants were native monolingual speakers of Greek, right-handed, and presented no history of neurodevelopmental disorders such as dyslexia, or prior neurological or psychiatric conditions, such as focal or diffuse brain damage including cerebrovascular disorders.

Enrollment of the persons with nfaPPA, as well as of controls, was consecutive from April 2023 to October 2023. For controls, exclusion criteria were: (a) diagnosis of psychiatric disorders, alcohol or drug abuse and dependence based on the criteria in the Diagnostic and Statistical Manual of Mental Disorders-5th edition (APA, 2013), (b) diagnosis of a neurological disease or any other medical condition and medical treatment regimen that may have affected cognitive performance, and (c) non-native speaker of Greek.

The participants were diagnosed with nfaPPA by a multi-disciplinary team, based on the individuals' language and clinical screening evaluation that fulfilled Gorno-Tempini et al.’s currently accepted criteria, including agrammatism in the individuals' language production, and impaired comprehension of syntactically complex sentences with preserved single-word comprehension and object knowledge abilities (Gorno-Tempini et al., 2011) (see Tables S1 and S2 in Supplementary Materials for the group means and the patients' individual scores in each screening test, respectively). None of the persons with nfaPPA had apraxia of speech, which is in line with previous research showing that languages having frequent consonant-vowel sequences, such as Italian and Greek, create fewer motoric challenges for a degenerating motor speech planning system as compared to languages with frequent consonant clusters, like English (Canu et al., 2020). All the persons with nfaPPA were assessed on their language abilities by a neurolinguist (the first author) with a 10-year experience in neurodegenerative language disorders, especially in persons with PPA, and the manifestation of their clinical symptoms. Neuropsychological assessment comprised the following tests: (i) the syntactic comprehension module of the Greek version of the Bilingual Aphasia Test (BAT) (Paradis & Keyagia, 1987), (ii) the Greek version of the Boston Naming Test-Short Form (BNT-SF) (Patricacou et al., 2007; Kaplan et al., 1983), (iii) the Greek auditory comprehension word discrimination test of the Boston Diagnostic Aphasia Examination-Short Form (BDAE-SF) (Goodglass & Kaplan, 1972; Peristeri & Tsapkini, 2011; Tsapkini et al., 2010), (iv) the picture version of the Pyramids and Palm Trees Test (PPT) (Breining et al., 2015), and (v) Digit forward (DF) and backward (DB) tests (Wechsler, 1997). The persons with nfaPPA were also administered the Greek version of the Mini Mental State Examination (MMSE) (Fountoulakis et al., 2000) as a measure of cognitive impairment, and the Raven Progressive Matrices (RPM) (Raven, 1983). Also, aphasia severity was assessed through the Aphasia Severity Rating Scale (ASRS) of the Greek BDAE-SF (Messinis et al., 2013). Scores on the ASRS range from 0 to 5, with 5 indicating very mild aphasic symptoms (‘minimal discernible speech handicap’) and 0 revealing very severe non-fluent aphasia (‘no useable speech or auditory comprehension’). Clinical assessment was performed by an experienced neurologist, and included the medical history with language concerns, brain imaging with magnetic Resonance Imaging (MRI), and cerebrospinal fluid (CSF) analyses. CSF was collected according to standardized operating guidelines (Teunissen et al., 2009).

The study protocols were approved by the local institutional review board and ethical committee of the Aristotle University of Thessaloniki (Approval number: 75092; Date of approval: 11/2022). Experimental data were collected following participants' formal written consent according to the Declaration of Helsinki. No part of the study procedures and analyses was pre-registered prior to the research being conducted.

Data collection was completed in three sessions for each participant. The time interval between sessions was one week. All tasks were administered in Greek.

### 2.2. Neuropsychological testing

#### 2.2.1. Nonverbal false belief task

Participants' false belief attribution abilities were assessed through a previously validated nonverbal task (Burnel et al., 2018; Forgeot & Ramus, 2011) run on E-Prime software (Schneider et al., 2012). The task comprises 19 different short video sequences illustrating a story. Each video consisted of four phases: a beginning (introducing the main agent of the scenario), a change phase (a change takes place in the nonverbal context that is either witnessed or not by the main agent), a suspense phase (the agent comes to the forefront, and is about to perform an action), and two possible ends, a correct and an incorrect one. Judging the likely end of the story required the participant either to attribute to the main agent a belief different from one’s own that would explain the action illustrated (Mentalistic end), or understand the outcome of a physical cause that was not related to the main agent’s belief (Mechanistic end) (see Burnel et al., 2018; Forgeot & Ramus, 2011 for examples of the task in the mentalistic and mechanistic condition). After the end of each video sequence, a question mark appeared on the center of the screen, and the participant was asked to respond whether the end was the most appropriate for the story, by pressing a [Yes] or a [No] button on a response device. Response accuracy (%) and reaction times (RT) on correct responses were recorded through E-Prime software.

### 2.3. Syntactic production: sentence repetition task

Sentence repetition performance was tested using the Greek version of the Sentence Repetition task that has been developed within COST Action IS0804 (Marinis & Armon-Lotem, 2015). The task includes 32 sentences of varying degrees of syntactic complexity, namely 12 simple clauses (Subject-Verb-Object sentences, and coordinate clauses), and 20 complex clauses consisting of 11 complement clauses and nine adjunct clauses (four adverbials and five relatives). Examples of complement and adverbial clauses in the sentence repetition task are cited below (see Andreou et al., 2020 for more examples of all sentences types in the task).

Complement clause

i nosokomes ipan oti i ptisi tu ɣiatru ehi kaθisterisi

the nurses said that the flight the doctor_GENITIVE_ has delay.

“The nurses said that the flight of the doctor is delayed.”

Adverbial clause

o ðaskalos piɣe kinimatoɣrafo eno protimuse na peksi kiθαrα

the teacher went cinema while preferred to play guitar.

“The teacher went to the cinema while he preferred to play the guitar.”

Words across sentences were matched for lexical frequency, length (number of syllables) and age-of-acquisition. All the sentences were pre-recorded by a female native speaker of Greek and had neutral intonation. The sound files were then incorporated in a PowerPoint presentation to ensure that all participants listened to the sentences in the same way. Each participant sat in front of a computer screen and listened to the sentences via headphones. Participants were asked to repeat exactly what they heard. Their responses were recorded with a digital voice recorder.

The participants' sentence repetition performance was scored for accuracy, and content to function word ratios. The accuracy scores were: 3 points if the sentence was repeated entirely verbatim, 2 points if the participant made one error, 1 point for two errors, and 0 points for three or more errors. The maximum accuracy score was 96 points (32 sentences, maximum of 3 points per sentence). Accuracy scores were computed separately for simple and complex clauses, as well for the two subcategories of complex clauses, i.e., complement and adjunct clauses. To compute the content to function word ratios, counts were made irrespective of whether the participants' sentence repetition output was grammatically correct or not.

### 2.4. Two digit-back updating task

The participants' updating abilities were measured using a two digit-back task (Smith & Jonides, 1999). A continuous stream of digits was viewed on the computer screen, and participants were required to decide, as fast as possible, whether the current digit matched the digit that was viewed two digits back. Digits were presented one at a time, with a stimulus duration of 500 msec, and an inter-stimulus interval of 2500 msec. Participants' responses were collected by pressing a pre-specified key (‘J’) on the keyboard. The 20 practice trials were immediately followed by a run of 60 trials. Across the 60 trials, 20 were the correct-hit trials. Correct-hits and errors, which consisted of false alarms and misses, were measured. The dependent variable was a composite accuracy score, calculated by subtracting the percentage of errors from the percentage of the correct-hits per participant. The task was designed with E-Prime software (Schneider et al., 2012).

### 2.5. Global-local attention shifting task

To assess the participants' ability to shift their attention we used a version of Navon’s global-local attention shifting task (Navon, 1977), which was run on a computer with E-Prime (Schneider et al., 2012). We compared Global-to-local and local-to-global attention shift costs (for more information on the materials and the procedure of the task see Peristeri et al., 2020).

In this task, four different shapes were used: circles, Xs, triangles and rectangles. Participants had to identify the number of lines required to formulate each shape, i.e., one line for circles, two for Xs, three for triangles, and four for rectangles, by pressing one of the four numbers (1, 2, 3, or 4) on the keyboard. Global shapes were constructed from either identical local shapes (congruent condition; e.g., a big triangle made up from smaller triangles) or different local shapes (incongruent condition; e.g., a big triangle made up from small Xs).

The task comprised three building blocks: (i) Global-No Shift, in which participants were asked to continuously respond to the global shape, (ii) Local-No Shift, in which participants were asked to continuously respond to the local shape, and (iii) Shift block, in which participants had to interchangeably respond to the global and local shape. Each target stimulus was presented on the screen until the participant responded. For the present study, we used data from the Shift block only. Local-to-global attention shifting cost in accuracy was calculated by subtracting accuracy scores in the local-to-global trials of the Shift block from accuracy in the global trials of the Global-No Shift block. Global-to-local attention shifting cost in accuracy was calculated by subtracting accuracy scores in the global-to-local trials of the Shift block from accuracy in the local trials of the Local-No Shift block.

### 2.6. Statistical analyses

To investigate whether the persons with nfaPPA would differ from controls in ToM, syntax, and EF abilities (*first research hypothesis*), linear mixed-effects models (LMEs) were used. In these models, Group was used as the predictor, while dependent measures included performances in nonverbal ToM, sentence repetition and EF, i.e., two digit-back updating and attention shifting. Specifically, dependent variables in the models comprised the following measures: accuracy and RT in the mentalistic and mechanistic trials of the false belief attribution task; total accuracy and noun-to-function word ratio in the sentence repetition task, as well as repetition accuracy in the simple and complex sentences, and accuracy in each of the two complex sentence-types, i.e., complement and adjunct sentences; accuracy in the two digit-back task; and global-to-local and local-to-global shifting costs in the global-local attention shifting task.

To understand the relationship between ToM performance, syntax and EF abilities in the nfaPPA group (*second research hypothesis*), we first ran simple regression models separately for each predictor, namely, syntax tested via sentence repetition (total accuracy, accuracy in simple, complex, complement, and adjunct clauses, content to function word ratio), EF (two digit-back accuracy, global-to-local and local-to-global attention shifting costs), MMSE and age. Accuracy in the mentalistic (Unseen Change) trials of the false belief task was the dependent variable. The significant regressions across the syntax and EF factors, and the ToM outcome provided appropriate evidence to continue with LMEs. Specifically, the predictors that have reached significance level (i.e., *p* < .05) were entered into LMEs to predict accuracy performance in the mentalistic trials of the false belief task for the nfaPPA group. Subjects were added to all the models as random effects, allowing for random intercepts. Coefficients and R^2^ of the best prediction model were used to determine the strengths of relationships among the variables involved in the model.

Finally, to better understand the direction and effect of the relationship between ToM performance, syntax and EF in the nfaPPA group, a standard mediation analysis was applied. In the analysis, ToM outcome (i.e., accuracy in the mentalistic trials of the false belief task) was the dependent measure, syntax (complement clauses) was the predictor, and EF was the mediating factor. The statistical significance of the relations considered in the mediation model was evaluated according to the R^2^ of the dependent construct (i.e., ToM) and the standardized path coefficient, i.e., the effect of the independent variable (i.e., syntax) on the outcome (i.e., ToM) controlling for the mediating variable (i.e., EF).

Statistical analyses were performed using JASP version .19.1.

## 3. Results

### Hypothesis (i). False belief attribution will be impaired in the persons with nfaPPA as compared to the language-unimpaired group, along with syntax and EF.

Table 1 presents the groups' performances in the main tasks of the study, namely, the nonverbal false belief attribution task, sentence repetition, two-digit back, and global-local attention shifting task.

The model fitted to the groups' accuracy in the ToM task found a significant Group effect for the mentalistic (i.e., Unseen Change) condition, such that the group with nfaPPA scored significantly lower in accuracy than the control group (estimate: −.17, SE: 2.12, *z* = −7.96, *p* < .001). On the other hand, there were no significant Group effects for accuracy in either the mechanistic trials (estimate: −.78, SE: 1.85, *z* = −.42, *p* = .675), or the RT in the mentalistic (estimate: 178.20, SE: 134.47, *z* = 1.32, *p* = .195) and mechanistic trials (estimate: 206.96, SE: 256.48, *z* = .80, *p* = .537) of the task.

In the sentence repetition task that tested syntactic abilities, the group with nfaPPA scored significantly lower than controls in overall accuracy (estimate: −23.11, SE: .77, *z* = −29.76, *p* < .001), as well as in complex clauses (estimate: −18.71, SE: .89, *z* = −20.91, *p* = .029), i.e., complements (estimate: −11.61, SE: .45, *z* = −25.58, *p* = .009), and adjuncts (estimate: −7.12, SE: .48, *z* = −14.76, *p* = .047). There was no significant Group effect for the simple clauses (estimate: −8.04, SE: 1.10, *z* = −7.29, *p* = .079) of the task. Also, the group with nfaPPA scored higher than controls in the noun-to-function word ratio (estimate: .19, SE: .03, *z* = 7.83, *p* < .001).

In the two-digit back task, the group with nfaPPA scored lower in accuracy than controls (estimate: −18.73, SE: 3.27, *z* = −5.73, *p* < .001).

Finally, in the global-local attention shifting task, the group with nfaPPA exhibited a significantly higher cost than controls when switching from global-to-local trials (estimate: −.09, SE: .02, *z* = −4.11, *p* < .001). The two groups did not differ in the cost they experienced when switching from local-to-global trials (estimate: −.02, SE: .03, *z* = −.52, *p* = .611).

### Hypothesis (ii). False belief attribution impairment will be predicted by syntax and EF performance in the persons with nfaPPA.

In the simple regression models, which were run separately for each predictor, R-squared (R^2^) and the root mean squared error (RMSE) were used as goodness-of-fit metrics. Two-digit back and accuracy in complement clause repetition had the highest R-squared values (mean R^2^ = .777 and .718, respectively; *p* < .001 for both predictors), while global-to-local, local-to-global and MMSE scores had lower R-squared values (mean R^2^ = .596, *p* < .001; mean R^2^ = .319, *p* = .021; and mean R^2^ = .276, *p* = .031, respectively). In the context of RMSE metrics, two-digit back and accuracy in complement clause repetition had the lowest values (RMSE = 5.989 and 6.741, respectively), while global-to-local, local-to-global and MMSE scores had higher RMSE values (RMSE = 8.063, 10.465, and 10.793, respectively). The associations between ToM and the rest of the predictors were not found to be significant (*p* = .147 for total accuracy in sentence repetition; *p* = .499 for accuracy in simple clauses; *p* = .072 for accuracy in complex clauses; *p* = .125 for accuracy in adjunct clauses; *p* = .130 for content to function word ratio; and *p* = .662 for age).

Variable selection in the LMEs was directed by the R^2^ and the RMSE metrics. Participants were added to all the models as random effects, allowing for random intercepts. Model development was finalized when no further improvement was observed. Among the LMEs, the one which fitted best the nfaPPA group’s ToM performance was the one that included complement clauses, two-digit back, global-to-local and local-to-global attention shifting costs as predictors (mean R^2^ = .833, RMSE = 5.175; see Table S3 in the Supplementary Materials for all the LMEs generated). The linear coefficients of the best prediction model show that performance in complement clauses was highly significant for the ToM performance in nfaPPA, since accuracy in the mentalistic (i.e., Unseen Change) trials of the false belief task increased for those persons scoring high in the repetition of complement clauses (Estimate: .711, *t* = 4.06, *p* < .001). Also, the persons with nfaPPA with higher scores in the digit task exhibited higher accuracy in the ToM task (Estimate: .424, *t* = 2.48, *p* = .01). Finally, the persons with nfaPPA that exhibited greater global to local shifting costs tended to be less accurate in the ToM task (Estimate: −1.102, *t* = −1.607, *p* = .045). The local to global shifting factor failed to reach significance (Estimate: −61.479, *t* = −1.117, *p* = .293).

The LMEs for the language-unimpaired group showed a marginally significant effect for the local-to-global cost only (Estimate: −71.09, SE: 6.81, *t* = −7.87, *p* = .06). The marginal effect stemmed from the fact that those who experienced greater costs when switching from local to global trials tended to be less accurate in the ToM task.

### 3.1. Mediation analysis results

**Fig. 1 –:**
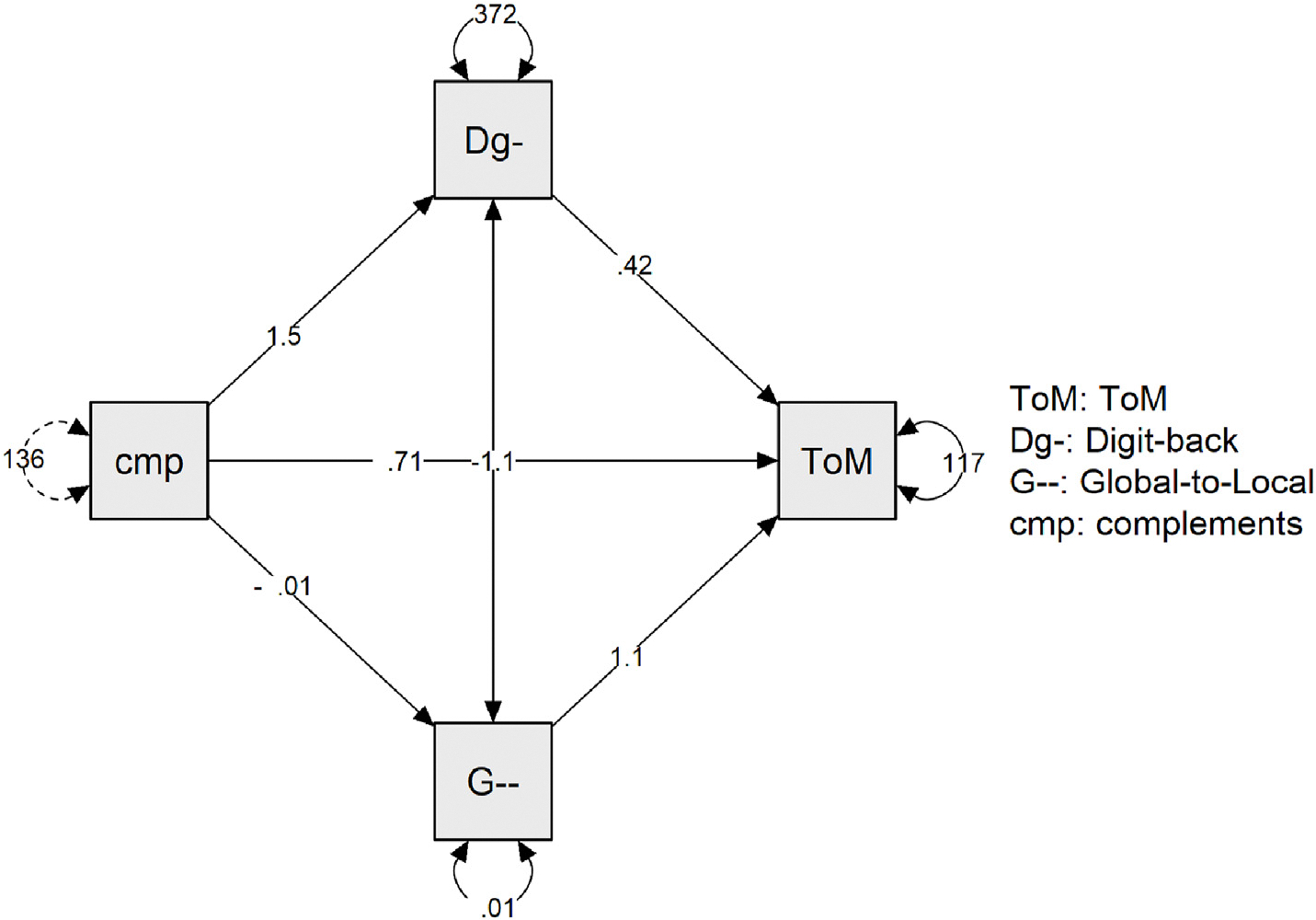
Mediation analysis: ToM, Complement clauses and EF (digit-back, global-to-local attention shifting cost) for the nfaPPA group.

A three-step mediation model (Baron & Kenny, 1986) was conducted on the relation between syntax (Complements) as the independent predictor variable, ToM as the dependent variable, and the two EF measures (global-to-local cost, digits back accuracy) (Iacobucci, 2012) as possible mediating variables in the nfaPPA group. The standardized values of the analysis are displayed in Fig. 1. The degree of possible mediation is also expressed using R^2^ values because coefficients of the independent variables in regressions do not respect additivity (Fairchild et al., 2009).

In Step 1: Syntax (complements) alone predicted R^2^_(syntax)_ = 72% of ToM’s variation [$\overset{ˆ}{c}=.71$ (*t* = 5.833, *p* < .001; when the mediators EF are not included)]. In Step 2: Syntax also significantly correlated with global-to-local cost [$\overset{ˆ}{a}=-.01$ (*t* = 1.540, *p* = .034)] and digit-back [($\overset{ˆ}{a}=1.5$ (*t* = 9.011, *p* = .001)]. In Step 3: when both Syntax (Complements) and the two EF measures were fit into the model as predictors of ToM scores, Syntax and EF predicted R^2^_(syntax & EF)_ = 83% of ToM’s variation, and Syntax was still a significant predictor [$\overset{ˆ}{b}=.36$ (*t* = 3.670, *p* < .001)]. Therefore, the indirect effect of Syntax on ToM is smaller than the total, though still significant. When syntax was removed from the latter model, EF predicted 64% of ToM’s variation. Based on the above, the effects of Syntax in explaining ToM’s variation in terms of R^2^ are:

- Total effect of Syntax: 72 %
- Direct effect of Syntax when controlling for (fixing) EF: 83%–64% = 19 %
- Indirect effect of Syntax other than through EF (Total – Direct): 72%–19% = 53%
- Percent of Total effect mediated by EF (Indirect/Total): 53/72 = 74 %

In summary, EF significantly but not fully mediated the relationship between Complement clauses and ToM performance in the nfaPPA group. We didn’t run a mediation analysis on the data of the language-unimpaired group because the LMEs for controls yielded no significant effects for syntax and EF on ToM.

## 4. Discussion

In the present study, we investigated for the first time (to our knowledge) impairments in false belief attribution through a nonverbal paradigm in persons with nfaPPA by comparing their performance to age- and education-matched language-unimpaired adults. We sought to determine whether the nfaPPA group would be impaired in ΤοМ (false belief attribution), and whether a ToM impairment would be associated with impairments in syntax and EF. We found that the persons with nfaPPA were impaired in the mentalistic condition of the false belief attribution task (requiring inferencing of the other’s belief), which tapped into ToM abilities, yet performed normally in the mechanistic condition (requiring causal naturalistic reasoning but no inferencing of the other’s belief). The persons with nfaPPA performed worse than controls in the syntactic (complex clause repetition for both complement and adjunct clauses), and EF tasks (two digit-back, global-local attention shifting task, especially in shifting from global to local stimuli), as expected. Importantly, syntactic (complement clauses), as well as EF (monitoring and updating, and attention-shifting abilities) impairments in nfaPPA significantly and independently predicted impairment in false belief attribution. These findings suggest that the persons with nfvPPA may have impaired performances in ToM due to impairments in basic non-social cognitive functioning, including syntax and EF.

The present study is the first to provide evidence of a deficit in ToM, and false belief attribution in particular, in PPA in the nonverbal modality. The false belief task in the current study proved to show sensitivity to nfaPPA persons' ToM deficit, since their performance was highly dissociable across the mentalistic and the mechanistic trials of the task. The fact that the persons with nfaPPA scored high and similarly to controls in the mechanistic condition, yet significantly lower than the control group in the mentalistic condition (see Table 1) across the same scenarios, implies that it was ToM impairment and not the complexity of the task itself that has driven ToM performance for the group with nfaPPA. Also, the finding that the effect of global cognition (as measured by the MMSE) failed to be included in the models significantly predicting ToM performance in the nfaPPA group (see Table S4 in Supplementary Materials) speaks in favor of a dissociation between ToM and global cognition as captured by tests such as the MMSE.

Another novel finding of the present study is that syntactic ability, measured through the repetition of complement clauses, significantly and independently predicted the false belief attribution performance in nfaPPA. The group performed significantly worse than controls in complex (complement & adjunct) clause repetition, yet it was only performance in complement clauses that was significantly related with the nfaPPA group’s false belief attribution abilities. These findings suggest a special affinity between complement clauses and false belief attribution, and are the first in neurodegenerative literature that support an isomorphism between syntax and ToM. Linguistic representations that are used to realize embedded complement clauses (e.g., John thinks that the weather is bad) have been claimed to be isomorphic to the cognitive structures needed to represent an inference, a claim or a belief (Tager-Flusberg, 2000). This isomorphism suggests a potential common cognitive substrate between false belief reasoning and embedded complement clauses that seems to be disrupted in the persons with nfaPPA. Notably, the current study revealed no association between both groups' performance in the ToM task and the repetition of non-complement adjunct clauses, i.e., adverbials and relatives. This suggests that not any type of sentence structure was taxing the ToM processing system of the persons with nfaPPA, as knowledge of adjuncts was not associated with the individuals' false belief attribution performance. Crucially, complement and adjunct clauses were matched in length, which precludes the possibility that the sentence-specific effects in the repetition task emerged as a result of the nfaPPA persons' short-term memory limitations. These findings suggest that a purely syntactic impairment predicts ToM impairment in false belief attribution independently from EF impairments in nfaPPA, even in nonverbal ToM task settings.

We should note, however, that even though the task that the current study used to assess ToM was nonverbal, one cannot preclude the possibility that ToM reasoning in the persons with nfaPPA was not verbalized through inner speech. The ToM task may have inflicted verbal introspections about the visual ToM scenarios and stimuli in the individuals, and these introspections might have been impaired in the persons with nfaPPA. There is evidence that inner speech is recruited when persons reflect on their own and others' thoughts, emotions and intentions, and that ToM partially relies on inner speech, because self-talk is one important way in which information about the self and others is communicated to the self (Carruthers, 2013). Relevant research has shown that inner speech can be preserved relative to spoken language in individuals with aphasia (Fama & Turkeltaub, 2020), and that inner speech reflects several important language processes in aphasia, including lexical retrieval and post-lexical representations (Fama et al., 2017, 2019). If the persons with nfaPPA employed inner speech to verbalize input in the nonverbal scenarios of the false belief task, one may assume that possible deficits in the nfaPPA persons' inner speech might have also contributed to their poor performance in the ToM task. More evidence of course is warranted to investigate if inner speech is impaired in nfaPPA and whether possible impairment in inner speech correlates with ToM reasoning in persons with nfaPPA.

The present study also found that the persons with nfaPPA scored significantly lower than controls in the monitoring and updating functions of the two digit-back task, and experienced greater costs than controls when shifting attention from global to local stimuli in the global-local attention shifting task, confirming previous findings on EF deficits in PPA [see Coemans et al. (2022)’s meta-analysis]. Crucially, both updating and attention shifting independently and significantly predicted false belief attribution in the persons with nfaPPA, suggesting that deficits in EF contribute to sociocognitive abilities such as ToM. On the other hand, only EF, and specifically attention-orienting abilities, were associated with ToM performance in controls. We should note that the EF tasks in the current study tapped into monitoring and updating functions within WM, and attention shifting rather than complex EF abilities, such as planning and cognitive flexibility or/and problem solving. Future research should address the possible contribution of complex EF abilities in the ToM performance of persons with PPA.

Finally, the mediation analysis demonstrated that although complex syntax (specifically, complement clauses) was the strongest predictor of false belief attribution in nfaPPA, this effect was mediated by WM, followed by nfaPPA persons’attention abilities. These results show that the ToM impairment in nfaPPA was consequent to impairments in syntax (particularly, complement clauses) and EF.

### 4.1. Limitations

We would like to acknowledge several limitations of the present study. The first limitation is the relatively small sample size (14 persons with nfaPPA). A power analysis using an increase of 768 msec (1 SD) in RT on the mentalistic trials of the ToM task found that the study would require 19 participants in the nfaPPA group to have 80% power to find this increase significantly different from controls at the *p* < .05 level. The number of the mentalistic (i.e., Unseen Change) items in the false belief task (19 trials per participant) was also limited. Furthermore, the inclusion of an extensive neuropsychological assessment would have given a clearer picture of the severity of cognitive dysfunctions in the persons with nfaPPA. Another main limitation relates to the lack of imaging or/and functional molecular measures for the persons with nfaPPA, which would have contributed to a more fine-grained clinical characterization of the study’s clinical sample. In the future, it would be important to compare ToM performance in persons with nfaPPA to other pathological groups, like individuals with semantic or logopenic variant PPA (Duval et al., 2012; Irish et al., 2014; Sakuta et al., 2021), or more common dementias such as amnestic Alzheimer’s disease, who have also been found to face difficulty with several aspects of ToM (Moreau et al., 2016; Sandoz et al., 2014). Finally, future studies should strive to correlate the functions assessed in the present study with MRI quantitative measures, which could further enhance existing knowledge of the topography of ToM processing (e.g., Calarge et al., 2003; Paunov et al., 2022) and the anatomical trajectories of ToM decline in persons with PPA.

## 5. Conclusions

The present study is the first to provide support for an impairment in ToM (in false belief attribution, in particular) in nfaPPA through a nonverbal paradigm, and the first to show that this impairment is predicted by non-social cognitive functioning abilities, including syntax (complementation) and EF (attention shifting, updating and monitoring). We believe that the most significant contribution of the present study is that it allows us to form hypotheses regarding possible contributions of syntax and EF to ToM abilities in nfaPPA, and ways to distinguish or even mitigate impairments in this domain. The evidence that syntactic and EF impairments predict ToM impairment in nfaPPA allows the intriguing hypothesis that if these syntactic or/and EF impairments can be treated, then ToM impairments may also be mitigated.

## Supplementary Material

1

2

Supplementary data

Supplementary data to this article can be found online at https://doi.org/10.1016/j.cortex.2025.03.012.

## Figures and Tables

**Table 1 -:** Groups’ mean accuracy scores, standard deviations (SD) and ranges in the ToM, sentence repetition, two-digit back, and global-local attention shifting task.

Tasks	Persons with nfaPPA		Controls	

**ToM task** – Mentalistic (Unseen Change)	Accuracy (%)	54.4 (12.6) 40–80	Accuracy (%)	88.1 (11.7) 70–100
	RT	2,492 (768) 1,303–4,046	RT	2,134 (213) 1,349–3,755
**ToM task** – Mechanistic	Accuracy (%)	86.4 (11.5) 70–100	Accuracy (%)	88.0 (10.0) 70–100
	RT	2,618 (462) 1040–3984	RT	2,145 (199) 724–4,943

**Sentence repetition**				
Total accuracy (max. score: 96)	38.9 (4.3) 32–46		85.1 (4.5) 76–92	
Simple clauses (max. score: 36)	22.3 (2.2) 18–26		30.9 (3.1) 27–36	
Complex clauses (max. score: 60)	16.6 (2.5) 12–20		54.2 (3.7) 47–60	
Complement clauses (max. score: 33)	5.4 (1.4) 4–10		28.7 (2.6) 23–33	
Adjunct clauses (max. score: 27)	11.2 (1.3) 8–13		25.5 (2.0) 22–29	
Noun to function word ratio	1.9 (.2) 1.6–2.1		1.4 (.1) 1.3–1.7	

**Two digit-back (%)**	13.9 (17.5) 30–40		41.4 (19.5) 20–90	

**Global-local attention shifting task**				
global-to-local attention shifting cost (%)	21.9 (14.7) 13–53		5.9 (9.3) 1–30	
local-to-global attention shifting task (%)	13.7 (10.3) 1–25		8.7 (13.1) 0–35	

Abbreviations: nfaPPA = non-fluent/agrammatic variant of primary progressive aphasia; ToM = Theory of Mind; RT = reaction times; max = maximum.

## Data Availability

All raw data supporting this research are publicly available: https://osf.io/b6cfp/
