# Supplementary material for: Theory of mind deficits in non-fluent primary progressive aphasia
Source: Cortex. Author manuscript; Available in PMC 2026 Jun 19. (PMC13281778; doi:10.1016/j.cortex.2025.03.012)
Supplement: 1 [file NIHMS2173851-supplement-1.pdf]

Scientific Transparency Report

Theory of mind deficits in non-fluent primary progressive aphasia

Corresponding author: Eleni Peristeri

Data

In this section authors indicate the public availability and access route(s) for all selected data categories as well as any barriers for data that cannot be fully shared.

Data category	Data type	Public availability	Availability barrier	Public access route	URL
Assessments - clinical sample	Raw	NO data are publicly available	Legal and ethical barrier	N/A - NO data are publicly available	N/A
Assessments - clinical sample	Processed	ALL data are publicly available	N/A - ALL data are publicly available	Data in repository (URL required)	https://osf.io/b6cfp/
Assessments - non-clinical sample	Raw	NO data are publicly available	Legal and ethical barrier	N/A - NO data are publicly available	N/A
Assessments - non-clinical sample	Processed	ALL data are publicly available	N/A - ALL data are publicly available	Data in repository (URL required)	https://osf.io/b6cfp/
Behaviour - clinical sample	Raw	NO data are publicly available	Legal and ethical barrier	N/A - NO data are publicly available	N/A
Behaviour - clinical sample	Processed	ALL data are publicly available	N/A - ALL data are publicly available	Data in repository (URL required)	https://osf.io/b6cfp/
Behaviour - non-clinical sample	Raw	NO data are publicly available	Legal and ethical barrier	N/A - NO data are publicly available	N/A
Behaviour - non-clinical sample	Processed	ALL data are publicly available	N/A - ALL data are publicly available	Data in repository (URL required)	https://osf.io/b6cfp/

Data category	Data type	Public availability	Restricted access route	Restricted access conditions
Assessments - clinical sample	Raw	NO data	Readers can never access the ‘restricted’ data	Readers can never access the ‘restricted’ data

Data category	Data type	Public availability	Restricted access route	Restricted access conditions
Assessments - non-clinical sample	Raw	NO data	Readers can never access the ‘restricted’ data	Readers can never access the ‘restricted’ data
Behaviour - clinical sample	Raw	NO data	Readers can never access the ‘restricted’ data	Readers can never access the ‘restricted’ data
Behaviour - non-clinical sample	Raw	NO data	Readers can never access the ‘restricted’ data	Readers can never access the ‘restricted’ data

Analytic Methods (Code)

In this section authors indicate the access route(s) for all types of analysis code that will be fully shared. All code will be publicly available and no barriers have been reported.

Code type	Public availability	Availability barrier	Public access route	URL (if applicable)
JMP code	ALL code is publicly available	N/A - ALL code is publicly available	Code in repository (URL required)	https://osf.io/b6cfp/

Research Materials

In this section authors explain how readers can access the research materials that are not publicly available and under what conditions. No materials will be publicly available.

Type of research materials	Public availability	Availability barrier	Public access route	URL (if applicable)
Code or software deployed via computer	NO materials are publicly available	Legal and technical barrier	N/A - NO materials are publicly available	N/A
Psychometric instrument(s) via computer or other electronic device	NO materials are publicly available	Legal and technical barrier	N/A - NO materials are publicly available	N/A
Psychometric instrument(s) in paper form	NO materials are publicly available	Legal barrier only	N/A - NO materials are publicly available	N/A
Clinical assessment tool(s) in paper form	NO materials are publicly available	Legal barrier only	N/A - NO materials are publicly available	N/A
Non-clinical task(s) or measurement(s) in paper form	NO materials are publicly available	Legal barrier only	N/A - NO materials are publicly available	N/A
Visual or auditory stimuli (e.g., still images, videos, sounds)	NO materials are publicly available	Legal barrier only	N/A - NO materials are publicly available	N/A

Type of research materials	Public availability	Restricted access route	Restricted access conditions
Code or software deployed via computer	NO materials	Readers can never access the 'restricted' materials	Readers can never access the 'restricted' materials
Psychometric instrument(s) via computer or other electronic device	NO materials	Readers can never access the 'restricted' materials	Readers can never access the 'restricted' materials
Psychometric instrument(s) in paper form	NO materials	Readers can never access the 'restricted' materials	Readers can never access the 'restricted' materials
Clinical assessment tool(s) in paper form	NO materials	Readers can never access the 'restricted' materials	Readers can never access the 'restricted' materials
Non-clinical task(s) or measurement(s) in paper form	NO materials	Readers can never access the 'restricted' materials	Readers can never access the 'restricted' materials
Visual or auditory stimuli (e.g., still images, videos, sounds)	NO materials	Readers can never access the 'restricted' materials	Readers can never access the 'restricted' materials

Design and Analysis Transparency

This article reports, for all studies, how the author(s) determined all sample sizes, all data exclusions, all data inclusion and exclusion criteria, and whether inclusion and exclusion criteria were established prior to data analysis.

Preregistration

The study procedures were not pre-registered.

The study analysis plans were not pre-registered.
