# Supplementary material for: Theory of mind deficits in non-fluent primary progressive aphasia
Source: Cortex. Author manuscript; Available in PMC 2026 Jun 19. (PMC13281778; doi:10.1016/j.cortex.2025.03.012)
Supplement: 2 [file NIHMS2173851-supplement-2.docx]

**Supplementary Materials**

**Table S1** Language and demographic information (mean, *SD* and range) of the group with nfaPPA and the control group.

|  | **Persons with nfaPPA** | **Controls** |
| --- | --- | --- |
| **Age of onset** | 61.5  (*4.2*)  53-73 | **-** |
| **Duration from onset to neuropsychological testing (years)** | 2.5  (*1.1*)  1-4 | - |
| **BAT-Syntactic comprehension** (max. score: 70) | 35.6  (*4.7*)  30-45 | 62.6 (*2.5*)  56-65 |
| **BNT-SF**  (max. score: 15) | 13.3  (*1.3*)  11-15 | 14.1 (*0.9*)  13-15 |
| **BDAE-SF**  **Word discrimination** (max. score: 16) | 15.2  (*0.4*)  15-16 | 15.7 (*0.6*)  14-16 |
| **BDAE-SF**  **Total score**  (max. score: 104) | 76.1  (*7.3*)  65-87 | - |
| **ASRS** | 1.9  (*0.7*)  1-3 | - |
| **RPM**  (max. score: 36) | 30.1  (*1.4*)  28-33 | - |
| **PPT**  (max. score: 52) | 47.9  (*2.6*)  45-52 | 50.6 (*1.1*)  49-52 |
| **DF**  (max. score: 9) | 3.6  (*1.3*)  2-6 | 6.9  (*0.7*)  5-8 |
| **DB**  (max. score: 9) | 1.9  (*1.2*)  0-4 | 5.4  (*1.6*)  2-8 |
| **MMSE**  (max. score: 30) | 24.8  (*1.5*)  22-27 | 28.1 (*1.0*)  27-30 |

Abbreviations: nfaPPA = non-fluent/agrammatic variant of primary progressive aphasia; BAT = Bilingual Aphasia Test; BNT-SF = Boston Naming Test-Short Form; BDAE-SF = Boston Diagnostic Aphasia Examination-Short Form; ASRS = Aphasia Severity Rating Scale; RPM = Raven Progressive Matrices; PPT = Pyramids and Palm Trees Test; DF = Digit Span Forward; DB = Digit Span Backward; MMSE = Mini Mental State Examination; max. = maximum; SD = standard deviation

**Table S2** Individual scores of the persons with nfaPPA in the screening tests.

| Person with nfaPPA | **BAT** | **BNT-SF** | **BDAE-SF**  **Word discrimination** | **BDAE-SF**  **Total score** | **ASRS** | **RPM** | **PPT** | **DF** | **DB** | **MMSE** |
| --- | --- | --- | --- | --- | --- | --- | --- | --- | --- | --- |
| P(erson)#1 | 32 | 12 | 15 | 79 | 1 | 30 | 45 | 6 | 0 | 24 |
| P#2 | 32 | 14 | 15 | 75 | 3 | 32 | 50 | 2 | 0 | 24 |
| P#3 | 40 | 11 | 15 | 73 | 3 | 28 | 50 | 5 | 2 | 26 |
| P#4 | 37 | 15 | 15 | 65 | 2 | 30 | 46 | 2 | 1 | 22 |
| P#5 | 39 | 14 | 16 | 72 | 2 | 29 | 47 | 4 | 3 | 23 |
| P#6 | 36 | 14 | 15 | 69 | 2 | 30 | 47 | 6 | 0 | 25 |
| P#7 | 39 | 13 | 15 | 82 | 3 | 28 | 50 | 4 | 3 | 24 |
| P#8 | 37 | 15 | 15 | 87 | 1 | 33 | 52 | 3 | 3 | 23 |
| P#9 | 4 | 14 | 15 | 71 | 2 | 30 | 47 | 4 | 3 | 25 |
| P#10 | 30 | 12 | 15 | 70 | 1 | 32 | 52 | 4 | 2 | 25 |
| P#11 | 30 | 13 | 16 | 83 | 1 | 31 | 50 | 3 | 2 | 27 |
| P#12 | 32 | 13 | 16 | 69 | 2 | 29 | 45 | 3 | 2 | 27 |
| P#13 | 30 | 11 | 15 | 85 | 2 | 29 | 45 | 2 | 2 | 26 |
| P#14 | 45 | 15 | 15 | 85 | 1 | 30 | 45 | 2 | 4 | 26 |

Abbreviations: nfaPPA = non-fluent/agrammatic variant of primary progressive aphasia; BAT = Bilingual Aphasia Test; BNT-SF = Boston Naming Test-Short Form; BDAE-SF = Boston Diagnostic Aphasia Examination-Short Form; ASRS = Aphasia Severity Rating Scale; RPM = Raven Progressive Matrices; PPT = Pyramids and Palm Trees Test; DF = Digit Span Forward; DB = Digit Span Backward; MMSE = Mini Mental State Examination

**Table S3** Summary statistics for the linear mixed effects model analyses of Theory of Mind performance in the nfaPPA group.

| **Model** | **Fixed Effects** | **R^2^** | **RMSE** |
| --- | --- | --- | --- |
| 1 | Accuracy in Complement clauses + Two digit-back | .798 | 5.699 |
| 2 | Accuracy in Complement clauses + Two digit-back + Global-to-local | .810 | 5.535 |
| 3 | Accuracy in Complement clauses + Two digit-back + Global-to-local + Local-to-global | .833 | 5.175 |
| 4 | Accuracy in Complement clauses + Two digit-back + Global-to-local + Local-to-global + MMSE | .814 | 5.465 |

*Note*. Each row contains the estimate of each covariate, the t-ratio, the associated Bonferroni corrected p-value, the R-squared (R^2^) and the root mean squared error (RMSE) metrics.
